# Inflammatory Markers in Patients With Major Depressive Disorder: A Prospective, Clinic-Based, Cohort Study From India

**DOI:** 10.7759/cureus.43059

**Published:** 2023-08-07

**Authors:** Sucharita Mandal, Mamidipalli Sai Spoorthy, Sangha Mitra Godi, Rachita Nanda, Bhaskar Mukherjee, Nihar Ranjan Mishra

**Affiliations:** 1 Psychiatry, All India Institute of Medical Sciences, Kalyani, Kalyani, IND; 2 Psychiatry, All India Institute of Medical Sciences, Bibinagar, Bibinagar, IND; 3 Psychiatry, All India Institute of Medical Sciences, Mangalagiri, Mangalagiri, IND; 4 Biochemistry, All India Institute of Medical Sciences, Raipur, Raipur, IND; 5 Psychiatry, Malda Medical College, Kolkata, IND; 6 Paediatrics, All India Institute of Medical Sciences, Kalyani, Kalyani, IND

**Keywords:** cohort, treatment, trait markers, depression, inflammation

## Abstract

Background

Patients with major depressive disorder have varying response rates to treatment. Multiple factors such as non-adherence, comorbidity, chronic stressors, and biological factors may be responsible for this variation. Inflammatory (pro and anti) markers have been well studied as a cause for depression, predisposing factors, and a consequence of depression. Among these, interleukins (ILs), interferons, C-reactive protein (CRP), and tumor necrosis factor-alpha (TNF-α) have been studied repeatedly. We conducted a pilot study to assess the levels of these inflammatory markers in patients with major depressive disorder. The specific objectives of this study were to compare and correlate changes in pro- and anti-inflammatory markers throughout different phases of depression, including pretreatment and posttreatment periods, and to evaluate the pattern of pro- and anti-inflammatory markers in patients who experienced remission or showed a positive response to treatment.

Methodology

This was a prospective, clinic-based, cohort study done for a period of one and a half years. Patients aged 18-65 years with depressive disorder per the International Classification of Diseases Tenth Edition and who scored more than 7 on the Hamilton Depression Rating Scale were included in this study. A total of 81 patients were recruited who were followed up till eight weeks after inclusion. A total of 31 patients completed the eight weeks of follow-up. Levels of IL-10 and TNF-α were assessed at baseline, two weeks, four weeks, and eight weeks of follow-up.

Results

This study tried to compare the levels of pro- and anti-inflammatory markers across pretreatment and various posttreatment phases of depression. Results showed that the levels of pro-inflammatory cytokine TNF-α increased from baseline till eight weeks of follow-up, and levels of IL-10 decreased from baseline till eight weeks of follow-up. However, these changes were not statistically significant.

Conclusions

This study supports the hypothesis that inflammatory markers can be trait markers of depression rather than the consequence or result.

## Introduction

In patients with depression, complete symptom remission is the ultimate goal as it has implications for functioning and quality of life [1]. However, approximately one-third of these patients fail to achieve remission despite multiple trials with different drug regimens [1], while another third experience relapse or recurrence of illness despite adherence to initially effective treatments. Treatment non-response in these subsets of patients can be attributed to various factors, including non-adherence, comorbid substance abuse, persistent chronic stressors, and underlying biological or genetic mechanisms. Biological factors contributing to variations in treatment response may involve hormones, inflammatory markers, reactive oxygen species, neurotrophic factors, and metabolic factors.

Existing data suggest a bidirectional relationship between inflammation and depression. Animal and clinical studies have identified an association between concentrations of pro-inflammatory cytokines, specifically interleukin (IL)-1β, IL-6, tumor necrosis factor-alpha (TNF-α), and depressive symptoms [2]. The pro-inflammatory state plays a role in depression through several mechanisms. It activates microglia and their secretomes, disrupts the blood-brain barrier via Th17 cells and IL-17, and increases the generation of reactive oxygen and nitrogen species which overwhelms cellular antioxidant pathways and causes damage to mitochondria and other cellular organelles. These mechanisms collectively impair the stress resilience of the brain and favor a depressive state [3,4].

Conversely, the role of anti-inflammatory cytokines in depression, such as one of the most important, IL-10, is also under investigation. Studies in animals have shown that mice lacking IL-10 expression exhibit decreased latency to immobility and prolonged immobilization time. Notably, the administration of IL-10 can reverse the depressive-like phenotype observed in IL-10-deficient animals [5]. The National Health and Nutrition Examination Survey revealed a high prevalence of heightened inflammation in individuals, with 47% of patients having a C-reactive protein (CRP) level above 3.0 mg/L and 29% having a CRP level above 5.0 mg/L [6].

Additionally, studies have examined interleukin and TNF-α levels in depression. A meta-analysis of 22 studies by Hannestad et al. investigated the effect of antidepressant medication treatment on serum levels of inflammatory cytokines and found that anti-depressant treatment did not impact TNF-α levels but reduced the levels of IL-1β and IL-6 [7]. Another study done by Zou et al. assessed serum levels of pro- and anti-inflammatory markers in 117 drug-naive patients with depression and 102 healthy controls, revealing increased levels of IL-1β, TNF-α, and IL-10, as well as lower levels of IL-8, in patients with depression [8].

To our knowledge, only a few studies conducted in India have examined the association between depression and inflammatory markers. Preliminary findings from these studies have demonstrated an association between depression and elevated pro-inflammatory markers [9]. However, data regarding anti-inflammatory markers and the changes in markers with antidepressant treatment remains inconclusive. Moreover, these studies mainly used TNF-α, IL-6, and IL-2 levels, with none focussing on IL-10. Therefore, it is necessary to identify these inflammatory markers in depression, particularly in the context of the Indian population. Considering the ethnic and genetic variations found in Western populations, identifying these markers may aid in developing treatment strategies tailored to Indian patients with depression [10].

Based on the aforementioned findings, we have undertaken a pilot study to assess the levels of these inflammatory markers in patients with major depressive disorder. The specific objectives of this study were twofold. First, we aimed to compare and correlate changes in pro- and anti-inflammatory markers throughout different phases of depression, including pretreatment and posttreatment periods. Second, we sought to evaluate the pattern of pro- and anti-inflammatory markers in patients who experienced remission or showed a positive response to treatment.

## Materials and methods

Study sample

We estimated the required sample size based on the mean differences of IL-10 and THF-α between patients with depression and healthy controls from the study by Carvalho et al. [11]. The mean (±SD) IL-10 values for patients and controls were 1.27 ± 0.35 and 1.64 ± 0.78, respectively, and the corresponding values for TNF-α were 2.36 ± 0.96 and 3.01 ± 1.33, respectively. We calculated the effect size (Cohen’s d) to be 0.60 and 0.56 for IL-10 and TNF-α, respectively. Based on the effect size of 0.56, alpha error probability of 5%, and power of 95%, the required sample size was estimated as 36. The sample size calculation was done using nMaster 2.0. Although we set out to have 36 participants with all completed follow-ups, we only had data for 31 participants who completed all follow-up assessments. For this, we had to recruit 81 participants. The attrition rate of 62% is explainable due to COVID-19-related restrictions, which were imposed during the data collection period.

A consecutive sampling technique was employed over a period of one and a half years to collect the study sample. Patients with major depressive disorder attending the psychiatry department of a tertiary care hospital who provided consent to participate in the study and fulfilled the inclusion criteria were recruited. The study included patients aged between 18 and 65 years diagnosed with major depressive disorder and recurrent depressive disorder according to the International Classification of Diseases-Diagnostic Criteria for Research (ICD-10 DCR), with scores above 7 on the Hamilton Depression Rating Scale (HAM-D) (including those with psychotic symptoms or suicidal ideation). Prior exposure to anti-depressant drugs was not a criterion for exclusion. Patients with bipolar depression, any other comorbid psychiatric diagnosis (excluding tobacco dependence syndrome), substance use disorder assessed using ICD-10 DCR, mental retardation or organic brain disorder, and any acute or chronic medical conditions with an inflammatory etiology were excluded from the study. The exclusion of acute or chronic medical conditions was done through history, physical examination, and screening of available lab investigations at the time of inclusion into the study.

Study design and procedure

This prospective cohort study was conducted over a period of one and a half years at the outpatient psychiatry department of a tertiary care hospital. Demographic and clinical details of all included patients were recorded using a standard sociodemographic profile format at baseline. The severity of depressive symptoms for all patients was assessed by authors SG and SM at baseline, two weeks, four weeks, and eight weeks using the HAM-D. Severity scoring was rated as mild (8-16) moderate (17-23), and severe (>24). Treatment adherence was assessed by obtaining a history from patients and cross-checking it with their caregivers. All patients were given pharmacotherapy alone with antidepressants, additional drugs such as benzodiazepines and antipsychotics are prescribed in a subset of patients. Treatment response was defined as a reduction of more than 50% in HAM-D scores. Levels of serum IL-10 (normal range = 4.28 ± 2.72 pg/mL) and TNF-α (normal range = non-detectable to 8.1 pg/mL) were measured in all patients at baseline, two weeks, four weeks, and eight weeks. The analysis of TNF-α and IL-10 was performed by enzyme-linked immunosorbent assay (ELISA)-IPL (Human TNF-α ELISA test kit and IPL-Human IL-10 ELISA test kit). Institutional ethics committee and research cell clearance was obtained before conducting the study.

Statistical analysis

All relevant data were recorded in a pre-designed case report format. The distribution of the data was assessed for normalcy using the Shapiro-Wilk test and found to be skewed. Therefore, the median with interquartile range was calculated for continuous data, and the categorical data were expressed as proportions. Statistical analysis was conducted using SPSS version 25.0 (IBM Corp., Armonk, NY, USA). Spearman’s correlation coefficient was used to estimate correlation statistics. Longitudinal time trend analysis was conducted with a two-tailed Friedman test. A p-value <0.05 was considered significant for all statistical purposes. Treatment response was characterized into categorical dependent variables (responders and non-responders).

## Results

The study started in August 2019 and continued for a period of one and a half years till December 2020. A total of 81 patients who met the inclusion and exclusion criteria provided informed consent and were recruited. However, only 48 patients returned for follow-up at two weeks. An additional seven patients were lost to follow-up at four weeks and 10 patients at eight weeks, resulting in a final sample size of 31 patients who completed the eight-week follow-up (study duration coincided with the onset of the first wave of COVID-19).

Table 1 shows the sociodemographic details of 81 patients included at baseline. The sociodemographic details of the 81 patients were more or less similar to that of the final 31 patients who were retained at eight weeks of follow-up.

**Table 1:** Sociodemographic details of the 81 patients included at baseline. Socioeconomic status: middle includes upper middle and lower middle class; lower includes upper lower and lower; and upper includes upper class from Kuppuswamy classification.

Variables	N (%)/Mean (SD)
Age (in years)	36.03 (11.50)
Body mass index (kg/m^2^)	27.54 (15.85)
Age of onset of illness	33.40 (11.48)
Gender	
Male	42 (51.8)
Female	39 (48.2)
Marital status	
Married	54 (66.7)
Unmarried	24 (29.6)
Widow	03 (3.7)
Educational status	
Primary school	23(28.4)
High school	11(13.6)
Intermediate	06(7.4)
Graduate	19(23.5)
Postgraduate	17(21)
Uneducated	5 (06.2)
Occupational status	
Employed	42(51.9)
Unemployed	39(48.1)
Socioeconomic status	
Upper	3 (3.7)
Middle	38 (46.9)
Lower	40 (49.4)
Religion	
Hindu	77 (95.1)
Muslim	3 (3.7)
Christian	1 (1.2)
Area	
Rural	35 (43.2)
Urban	46 (56.8)

Table 2 shows the sociodemographic profile of the 31 patients. The mean age of the sample was 36.03 years (SD = 11.71). Most patients were married, unemployed, only had a primary school education, and were of low socioeconomic status. The mean weight and body mass index (BMI) of the sample were 60.7 kg (SD = 11.67) and 31.4 kg/m^2^ (SD = 21.51), respectively.

**Table 2:** Sociodemographic details of the study sample who completed the eight-week follow-up. Socioeconomic status: middle includes upper middle and lower middle class; lower includes upper lower and lower; and upper includes upper class from Kuppuswamy classification.

Variables	N (%)	Median (IQR)
Age (years)		36.0 (26.0, 46.0)
Body mass index (kg/m^2^)		23.3 (21.0, 29.1)
Age of onset of illness (Years)		30.0 (25.0, 42.0)
Gender		
Male	16 (51.6)	
Female	15 (48.4)	
Marital status		
Married	11 (35.5)	
Unmarried	19 (61.3)	
Widow	01 (03.2)	
Educational status		
Primary school	11 (35.5)	
High school	05 (16.1)	
Intermediate	03 (09.7)	
Graduate	04 (12.9)	
Postgraduate	06 (19.4)	
Uneducated	02 (06.4)	
Occupational status		
Employed	17 (54.8)	
Unemployed	14 (45.2)	
Socioeconomic status		
Middle	13 (41.9)	
Lower	18 (58.1)	
Religion		
Hindu	29 (93.6)	
Muslim	02 (06.4)	
Area		
Rural	14 (45.2)	
Urban	17 (54.8)	

Table 3 shows the clinical characteristics and treatment-related variables of the patients. The majority of the sample had severe depression (HAM-D >24), with a median number of previous episodes of 1.0 (interquartile range (IQR) = 1-2), a median duration of illness of 1.5 (IQR = 0.5-3) years, and an average duration of the current episode of 6.0 (IQR = 2-12) months. Among the patients who completed the eight-week follow-up, 64.6% (20) were receiving escitalopram, and dose adjustments were made in 77.4% (24) of patients during the eight-week follow-up period. The mean starting dose of escitalopram was 10.5 mg/day, and the mean dose increase from baseline to eight weeks was 5.5 mg/day.

**Table 3:** Clinical characteristics and treatment-related variables. HAM-D scoring: 8-16, mild; 17-23, moderate; >24, severe depression.

Variables	N (%)	Median (IQR)
Total duration of illness (years)		1.5 (0.5, 3.0)
Duration of current episodes (months)		6.0 (2.0, 12.0)
Number of episodes		1.0 (1.0, 2.0)
Categories of depression		
Mild depression	06 (19.4)	
Moderate depression	09 (29.0)	
Severe depression (with and without psychotic symptoms)	16 (51.6)	
Status of past psychotic illness		
Absent	29 (93.6)	
Present	02 (06.4)	
Status of past suicidal behavior		
Absent	26 (83.9)	
Present	05 (16.1)	
Status of past hospitalization		
Absent	29 (93.6)	
Present	02 (06.4)	
Status of past treatment history		
Absent	14 (45.2)	
Present	17 (54.8)	
Types of drugs used in the past		
No	16 (51.6)	
Selective serotonin reuptake inhibitors	07 (22.6)	
Serotonin and norepinephrine reuptake inhibitors	03 (09.7)	
Antipsychotic	03 (09.7)	
Antidepressant with a mood stabilizer	01 (03.2)	
No data available	01 (03.2)	
Types of drugs used currently		
Escitalopram	20 (64.5)	
Others	11 (35.5)	
Baseline dose (mg)		10.0 (10.0, 25.0)
Dose at 2 weeks (mg)		15.0 (10.0, 40.0)
Dose at 4 weeks (mg)		20.0 (15.0, 60.0)
Dose at 8 weeks (mg)		20.0 (20.0, 60.0)
Dose change between baseline and 2 weeks (mg)		5.0 (0.0, 7.5)
Dose change between baseline and 4 weeks (mg)		10.0 (0.0, 15.0)
Dose Change between Baseline & 8 weeks (mg)		10.0 (5.0, 25.0)
Polytherapy		
No	25 (80.6)	
Yes	06 (19.4)	
Family history of psychiatric illness		
No	18 (58.1)	
Yes	13 (41.9)	
Response to treatment		
No	09 (29.0)	
Yes	22 (71.0)	

About 71% (22 of 31) of patients who were followed up had more than a 50% reduction in HAM-D scores. In the current study, five (16.1%) patients exhibited suicidal behavior in the past, and 17 (54.8%) had a previous treatment history for depression.

**Figure 1:**
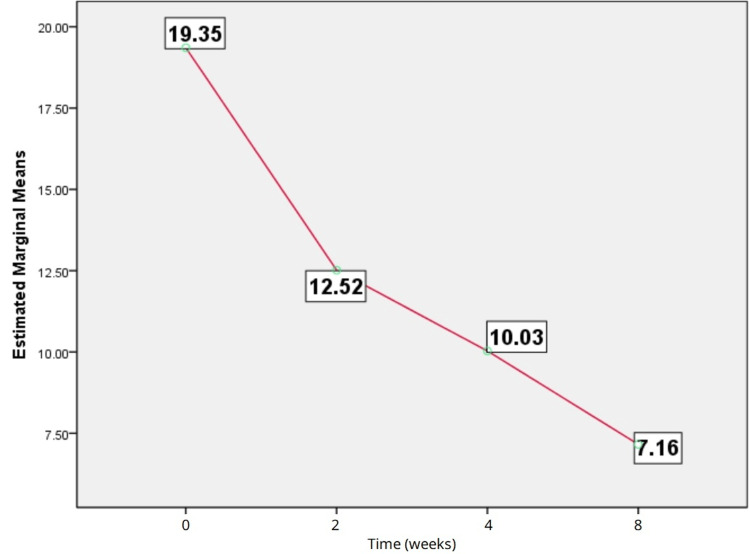
Estimated means of Hamilton Depression Rating Scale scores.

The results of the HAM-D scores showed improvement in depressive symptoms from baseline at two weeks, four weeks, and eight weeks of treatment. Friedman test revealed a significant difference between baseline, two weeks, four weeks, and eight weeks of follow-up (chi-square = 73.37, df = 3, p < 0.0001), as shown in Figure 1. Wilcoxon signed rank test showed a significant difference (p < 0.05) in HAM-D scores at several timelines (baseline vs. two weeks, two weeks vs. four weeks, four weeks vs. eight weeks, baseline vs. eight weeks).

**Figure 2:**
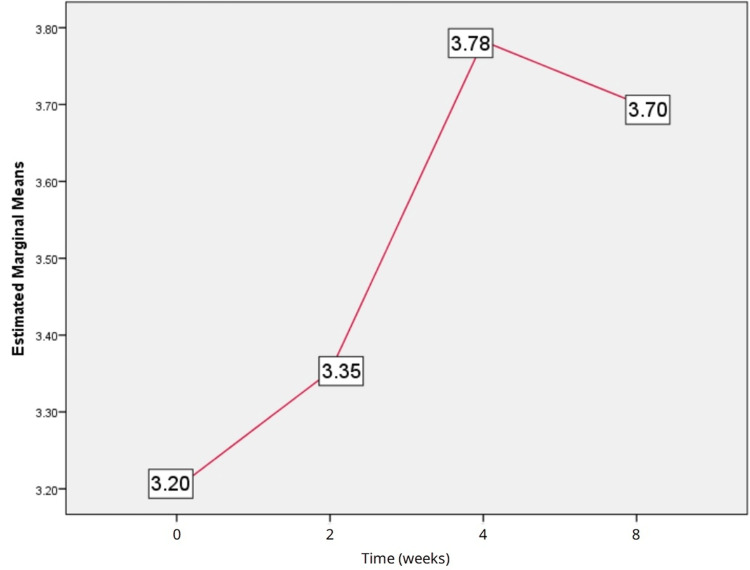
Estimated means of tumor necrosis factor-alpha.

Analysis of TNF-α levels (pg/mL) indicated an increase from baseline at two weeks, four weeks, and eight weeks of treatment. Friedman test results found significant differences between baseline, two weeks, four weeks, and eight weeks of follow-up (chi-square = 8.650, df = 3, p = 0.034), as shown in Figure 2. Wilcoxon signed rank test showed a significant difference (Z = -2.410, p = 0.016) in the levels of TNF-α between baseline and eight weeks of follow-up but not with other timelines (baseline vs. two weeks, two weeks vs. four weeks, four weeks vs. eight weeks).

**Figure 3:**
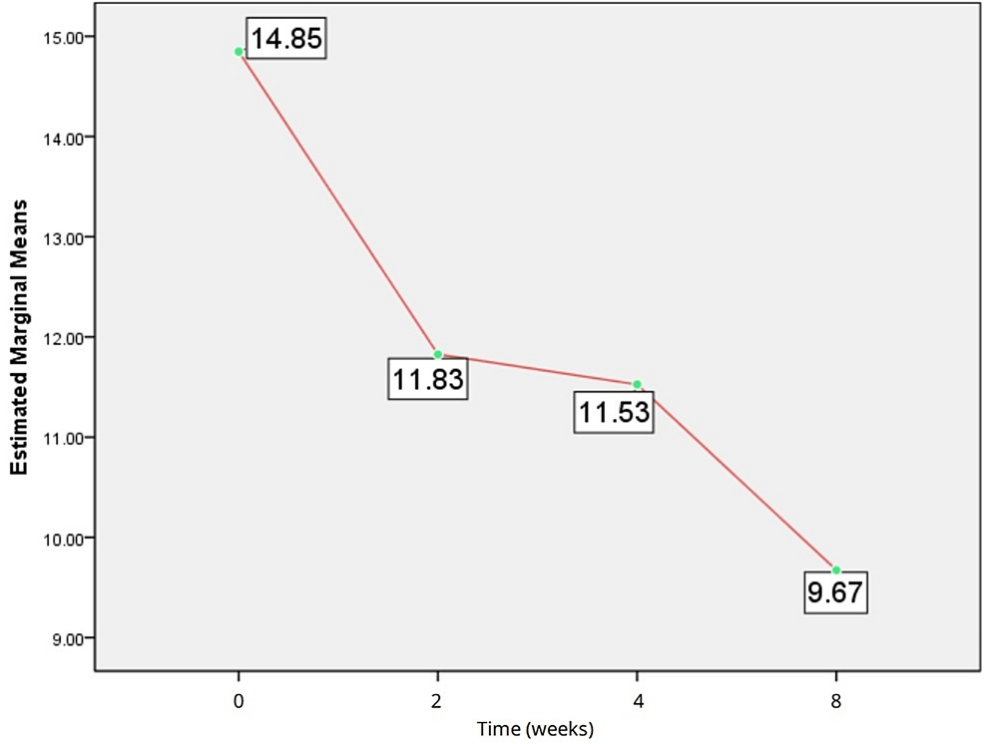
Estimated means of interleukin-10.

Analysis of IL-10 levels (pg/mL) revealed a decrease from baseline at two weeks, four weeks, and eight weeks of treatment. However, the Friedman test showed no significant difference between baseline, two weeks, four weeks, and eight weeks of follow-up (chi-square = 3.078; df = 3, p = 0.380), as shown in Figure 3. Wilcoxon signed rank test showed no significant difference in the levels of TNF-α between different timelines of follow-up (baseline vs. two weeks, two weeks vs. four weeks, four weeks vs. eight weeks, baseline vs. eight weeks; p > 0.05).

Table 4 presents the results of the correlation analysis conducted to examine the mean change in HAM-D scores, mean change in TNF-α levels, and mean change in IL-10 levels from baseline to eight weeks of follow-up. Of note, the analysis did not reveal any significant correlation between these variables. There was a strong negative correlation between serum TNF-α (pg/mL) and serum IL-10 (pg/mL) at baseline (r = -0.679, p < 0.001) and a weak negative correlation at two weeks of follow-up (r = -0.534, p < 0.05). However, at four weeks and eight weeks of follow-up, the correlation was not found to be significant (r = -0.084, p > 0.05; r = -0.070, p > 0.05).

**Table 4:** Correlation between HAM-D scores and TNF-α and IL-10 levels. HAM-D: Hamilton Depression Rating Scale; TNF-α: tumor necrosis factor-alpha; IL-10: interleukin 10

Mean change	Mean TNF-α change (r/p)	Mean IL-10 change
Mean HAM-D change from baseline to 8 weeks	-0.013 (0.943)	0.223 (0.227)
Mean TNF-α change	1.00	0.283 (0.123)

## Discussion

This study aimed to compare the levels of pro- and anti-inflammatory markers throughout different phases of depression from pre-treatment to post-treatment. Our findings revealed that there was a significant increase in the levels of the pro-inflammatory cytokine TNF-α from baseline to the eight-week follow-up. These results are consistent with previous studies [7,12,13]. However, contrasting results have been reported in other studies [14,15], which found that TNF-α levels were significantly higher in depressed patients compared to controls at baseline and that these levels were significantly reduced after antidepressant treatment at follow-up.

The role of IL-10 in regulating inflammation is hypothesized to be through its modulation of the hypothalamic-pituitary-adrenal axis and the ability to regulate pro-inflammatory cytokines. [5,16] The anti-inflammatory marker IL-10 levels in this study were not significantly different between baseline and post-treatment levels at eight weeks of follow-up. Still, the levels were high at baseline, as in the study by Zou et al. and Dahl et al. which showed a declining trend over time [8,17]. This is inconsistent with earlier animal and human studies which found a lack of expression of the IL-10 gene or low levels of IL-10 was associated with depression and administration of IL-10 reverses depression [5,18,19].

As in most studies and treatment guidelines for the treatment of depression, the 6-12-week time frame is taken as an adequate trial for antidepressants as the changes in neurotransmitter receptor sensitivity correlate with the above-mentioned time period and probably the changes in the levels of cytokine or inflammatory levels also takes more than 6-12 weeks to start reducing to the baseline state [20]. This is also consistent with the study by Dahl et al., which found that although IL-10 and other pro-inflammatory markers decreased significantly from the baseline, the decline was not observed at the 12-week follow-up but at two months after full remission. This indicates the longer time frame after remission for normalization of the inflammatory state and probably much longer follow-up duration would have resulted in different results [17,21]. In the case of our study, as it was a pilot trial, the study follow-up duration was restricted to eight weeks. During these eight weeks, dropout rates were also significantly higher (due to the first wave of the COVID-19 pandemic).

This study found that either symptom severity or response to treatment in depressive symptoms did not significantly change the levels of IL-10 but levels of TNF-α have significantly changed. This finding is consistent with previous studies that reported a significant positive correlation between TNF-α and the severity of depression, such as those conducted by Zou et al. and Fan et al. [8,22].

The other hypothesis is that the inflammatory process can be a trait marker of an individual which was consistently present during the normal state of an individual that further predisposes the individual for depression in the presence of other biological and psychosocial factors rather than a marker of the acute state or chronic state of illness that increases only during exacerbation of illness is one probable explanation [7,23-25]. Hence, the role of inflammatory markers as biomarkers of treatment response was still inconclusive and can be an area of interest for further research.

Our study has several limitations that should be acknowledged. First, the small sample size limits the generalizability of the findings. Although our initial sample was 81 cases, we could not follow up the entire sample due to losing participants in view of the COVID-19 pandemic and related restrictions. The lockdown measures and disrupted outpatient department services posed significant barriers to sample collection. Future studies should aim to include larger sample sizes, especially in the context of India, where research on this topic is lacking. Additionally, including other pro- and anti-inflammatory markers beyond TNF-α and IL-10 could provide a more comprehensive understanding of the inflammatory processes in depression. Moreover, incorporating a healthy control group would enable better comparisons of pro- and anti-inflammatory markers between depressed and normal healthy individuals. Furthermore, our study could not assess the potential confounding effect of medication on the levels of inflammatory markers.

## Conclusions

This study tried to assess inflammatory markers as the biomarkers of treatment response in depression. Although a significant decrease in HAM-D scores and a significant increase in pro-inflammatory marker (TNF-α) was found from baseline to eight weeks post-treatment, no significant difference was found in anti-inflammatory (IL-10) marker levels across pre-treatment and various post-treatment phases of depression. These findings suggest that treatment response does not necessarily imply a decrease in inflammation within eight weeks of follow-up in the Indian context. The relationship between the severity of depressive illness and cytokine levels may depend on other factors, such as chronicity, the normal inflammatory state of an individual, or possibly even influenced by other genetic factors. Even though IL-10 was not found as a marker for treatment response, TNF-α was found to significantly increase at the end of follow up paving the way for future research with a larger sample size.
